# Buses, Cars, Bicycles and Walkers: The Influence of the Type of Human Transport on the Flight Responses of Waterbirds

**DOI:** 10.1371/journal.pone.0082008

**Published:** 2013-12-18

**Authors:** Emily M. McLeod, Patrick-Jean Guay, Alice J. Taysom, Randall W. Robinson, Michael A. Weston

**Affiliations:** 1 Applied Ecology Research Group and Institute for Sustainability and Innovation, College of Engineering and Science, Victoria University, Melbourne, Australia; 2 College of Health and Biomedicine, Victoria University, Melbourne, Australia; 3 Centre for Integrative Ecology, Faculty of Science, Engineering and the Built Environment, School of Life and Environmental Sciences, Burwood, Australia; Institut Pluridisciplinaire Hubert Curien, France

## Abstract

One way to manage disturbance to waterbirds in natural areas where humans require access is to promote the occurrence of stimuli for which birds tolerate closer approaches, and so cause fewer responses. We conducted 730 experimental approaches to 39 species of waterbird, using five stimulus types (single walker, three walkers, bicycle, car and bus) selected to mimic different human management options available for a controlled access, Ramsar-listed wetland. Across species, where differences existed (56% of 25 cases), motor vehicles always evoked shorter flight-initiation distances (FID) than humans on foot. The influence of stimulus type on FID varied across four species for which enough data were available for complete cross-stimulus analysis. All four varied FID in relation to stimuli, differing in 4 to 7 of 10 possible comparisons. Where differences occurred, the effect size was generally modest, suggesting that managing stimulus type (e.g. by requiring people to use vehicles) may have species-specific, modest benefits, at least for the waterbirds we studied. However, different stimulus types have different capacities to reduce the frequency of disturbance (i.e. by carrying more people) and vary in their capacity to travel around important habitat.

## Introduction

‘Disturbance’ is the disruption of the normal activity or physiology of wildlife, such as birds, in the proximity of an agent such as a person or vehicle. In some circumstances disturbance is regarded as a conservation problem [1]–[3]. The classic mechanistic model of bird disturbance involves an external ‘stimulus’ (e.g. a person), and a ‘response’ on the part of the bird (e.g. escape), with various internal (e.g. body weight, species) and external (e.g. speed of approach) influences mediating the response [4], [5].

While great variation in the form and intensity of escape responses occurs, including substantial variation within species, several general principles regarding animal escape have been elucidated [4]. One of the basic principles which has been described regarding bird disturbance by humans is that the nature and behaviour of the stimulus influences the probability and extent of response [6]–[8]. For example, walkers may evoke responses of shorebirds at different distances than those evoked by dog walkers or joggers [9]. Different stimuli are often associated with multiple cues (visual, auditory or olfactory) and birds may respond to these cues separately as well as holistically; for example, birds may respond to a recording of a barking dog [10]. The behaviour of stimuli may also influence responses, for example, the unpredictable and rapid movements of unleashed dogs may explain the greater responses of birds to unleashed rather than leashed dogs [6], [11].

Anthropogenic stimuli come in many shapes and forms, but few studies actually examine the responses of birds to different stimuli ‘likely’ to occur in areas of natural significance ([12]–[16], but see [17]). An understanding of which stimuli are associated with more frequent or intense responses could aid planning and promote coexistence between humans and wildlife. An example of this is areas of high natural significance (i.e. those harbouring substantial biodiversity) and the question as to how humans should be able to use such areas. Humans could be permitted on foot or by bicycle (potentially representing low acoustic cues). Alternatively, people could access such areas in vehicles, such as cars or buses (permitting fewer vehicles because they have higher carrying capacities but representing larger, noisier stimuli). In essence, these choices represent a potential management continuum of self-directed (walking, cycling, some vehicles) to organised ecotourism (some vehicles but especially buses).

Given that human presence can be detrimental to wildlife such as birds, the management of human access into sensitive natural areas is critical [4]. A common way to manage human disturbance in sensitive areas involves the establishment of buffer/exclusion zones (attempts to completely exclude people are not always effective e.g. [18]). Ideally, the size of buffer zones is determined using Flight Initiation Distance (FID), the distance at which birds responds to various stimuli [19]. Although the responses of birds differ markedly between stimuli, it has been suggested that available FIDs are dominated by those evoked by single walkers [4]. However, this has not been tested.

This study aims to: 1) determine if there is a bias in the literature to reporting more FIDs evoked in response to a single walker; and 2) examine FIDs evoked by five different (but commonly occurring) stimuli: single walker, a group of (three) walkers, bicycle, car, and bus. We control for a range of other factors by conducting the study at a site which currently experiences relatively low levels of human presence compared with publically accessible sites nearby [9]. Managers are seeking advice on the least-disturbing human presence for birds at this site (W. K. Steele pers. comm.).

## Methods

### Literature search

We performed a search in Google Scholar 12^th^ October 2012 using the keywords “bird” and “flight initiation distance” (see Figure S1). The keywords “bird” and “flush distance” were used in an additional search performed in the same database (14^th^ January 2013). These searches returned a total of 695 papers. Of these, only the 100 studies that measured FID in birds were considered further. The stimuli which had been used in each study were determined and details of each paper were noted. For each study, we extracted the stimuli used and the species studied. For studies comparing multiple stimuli within species, we recorded the comparisons made and whether significant differences were reported.

### Fieldwork

Field work was conducted at the Western Treatment Plant (WTP), Werribee, near Melbourne, Victoria, Australia (38°1′S, 144°34′E). The Ramsar-listed WTP holds internationally significant numbers of many waterbird species and is a renowned birdwatching site [20], [21]. Access to the plant is restricted; visitors are required to obtain a permit and register each visit. The common birdwatching areas of the WTP are comprised of various ponds and lagoons and the coastline, all of which are easily accessible via car or foot from the roads and paths that run throughout the plant, usually between every pond. In addition to the birdwatchers and workers in cars or on foot, bus tours of the WTP are often conducted. The waterbirds at the WTP are thus exposed to some human activity, less than that evident in unrestricted areas such as urban parks [9].

### Measuring flight-initiation distances

We collected FIDs for 39 waterbird species between September 2011 and February 2012. All fieldwork was conducted between 0730 and 2100 hours, and as is customary and practical, only when it was not raining. We presented five types of stimuli to waterbirds within the WTP: single walker (1.4 ms^−1^), three walkers (1.0 ms^−1^), bicycle (2.0 ms^−1^), car (2.8 ms^−1^) and bus (2.8 ms^−1^). A stimulus type was randomly selected for each fieldwork day. For each stimulus type, FID was assessed by moving towards the focal bird at a constant pace. While approach speeds can influence FIDs [22] we used approach speeds which were typical of the stimuli being tested; our aim was to mimic realistic behaviour of each stimulus type. During the approach the observer/s were silent and made no sudden body movements. The distance at which we started an approach was recorded as the Starting Distance, and was maximised i.e. we used the longest Starting Distance possible [5], [23]. The distance at which the bird walked, swam, dived, or flew away in response to the approach was recorded as the FID. Approaches were only included if the bird's response was determined to occur as a result of the approach. When a flock was approached, the FID was taken from the point at which the first individual showed a response to the approach. An approach was abandoned if it was unclear whether the bird was responding to the observer or to another potential stimulus, such as a bird of prey. Depending on the target bird's original location, we approached either directly or tangentially. For tangential approaches, we minimised bypass distance and bypass distance was thus reasonably modest (29.4±1.0 m [mean ± SE]; 493 tangential approaches. All distances were measured using a laser rangefinder.

All approaches were made by EMM and AJT. For all walking and bicycle approaches the observers wore standard clothes (dark pants and a dark long-sleeved top). In all bicycle approaches observers also wore a bicycle helmet. All approaches were conducted on non-breeding adult waterbirds and only single-species flocks were approached. We attempted to avoid resampling individuals by closely monitoring where birds flushed to after an approach, before moving on to the next site. We present all raw FID and Starting Distance data, following the recommendation of Weston *et al.* [4].

### Statistical analysis

For tangential approaches, FID was calculated as the Euclidian distance between the observer and the subject at the time escape behaviour was initiated by taking into account the bypass distance, the minimum distance between the focal bird and the path of the observer [24]. FID did not differ between tangential and direct approaches (*F*_1,403_ = 0.878; *P* = 0.349) so data for both approach types were pooled for further analysis.

We were not able to measure FID against all stimuli for all species because of the sample sizes achieved, an artefact of locating birds in appropriate locations and manoeuvring stimuli to enable useful data collection. We therefore restricted our statistical analyses to four species (Australian shelduck *Tadorna tadornoides*, black swan *Cygnus atratus*, chestnut teal *Anas castanea*, and little pied cormorant *Microcarbo melanoleucos*). For these species we obtained at least five FID estimates per stimulus. We used a General Linear Model (GLM) to investigate the effect of species, stimulus type, and their interaction, and Starting Distance on FID using data from those four species. To test for potential differences in the relationship between Starting Distance and FID between stimuli and between species, we included two-way interactions, i.e. between Starting Distance and stimulus type and Starting Distance and species. We further used GLMs to compare responses between stimulus for all species where at least two stimuli had sample sizes of five or more (n = 12 species). Estimated Marginal Means (EMM) were calculated from these GLMs and two-tailed post hoc tests were performed using the EMM standard errors to compare FID between stimuli within species. All distances were Log_10_ transformed prior to analyses. Summary statistics are presented as mean ± standard deviation.

### Permissions

Data were collected under Deakin University Animal Ethics Committee Permit A48/2008, Victoria University Animal Ethics Committee Permit AEETH 15/10, National Parks Permit 10004656, DSE Scientific Permits Nos 10004656 and 10005536, and Western Treatment Plant Study Permit SP 08/02. Techniques used were non-invasive, and all were under permit and ethics approval.

## Results

The 100 studies located described FIDs evoked by 1.17±0.51 stimulus types per paper (1–4). Most studies reporting FIDs in birds only reported estimates derived from approaches by single walkers (73%; Table 1). The diverse mixture of species and stimuli tested, and the unbalanced nature of the sample, meant statistical comparisons between stimuli were unsuitable. Only 13% of studies, involving 44 species, compared more than one stimulus type. These studies report a total of 70 comparisons of FID between any two given stimulus types (Table 2).

**Table 1:** Papers (n = 100) which provide data on Flight-Initiation Distance (FID) in birds evoked by various stimuli.

Stimulus	Number (percentage) of studies	Source	Number of species*
Single walker	82 (82%)	[7]–[9], [23], [25], [28], [30]–[105]	392
Motorised boat	8 (8%)	[27], [29], [106]–[111]	33
Multiple walkers	8 (8%)	[8], [27], [29], [31], [32], [112]–[114]	21
Jogger/runner	2 (2%)	[9], [25]	9
Dogs on and off leash	4 (4%)	[9], [25], [39], [52]	14
Non-motorised boat (canoe/raft)	4 (4%)	[27], [115]–[117]	4
Car, 4×4	3 (3%)	[29], [30], [118]	75
Truck	1 (1%)	[29]	3
Airboat	1 (1%)	[119]	13
Ship	1 (1%)	[120]	4
Jet ski	1 (1%)	[110]	23
Helicopter	1 (1%)	[121]	1
Radio-controlled vehicle	1 (1%)	[67]	1

We present the number of studies reporting FIS data for each stimulus as well as the number of unique species for which data is presented.

^*^excludes one reference [104] for which no species list was available.

**Table 2:** Papers which report comparisons of Flight-Initiation Distances (FID) between various stimuli.

Comparison	Sources	Number of species compared	FID Outcome (number of species comparisons)^1^
SW vs MW	[8], [31], [32]	4	MW>SW (1)
			MW = SW (3)
SW vs Jogger	[9], [25]	9	Jogger>SW (4)
			Jogger = SW (5)
SW vs Dog	[9], [25], [39]	12	Dog>SW (5)
			SW>Dog (2)
			Dog = SW (7)
Jogger vs Dog	[9], [25]	9	Jogger>Dog (2)
			Dog>Jogger (2)
			Jogger = Dog (5)
Car vs Truck	[29]	2	Car>Truck (1)
			Truck>Car (1)
Car vs SW	[30]	6	Car>SW (1)
			SW>Car (2)
			Car = SW (3)
MB vs NMB	[27]	1	MB = NMB (1)
MB vs Jet ski	[110]	16	Jet ski>MB (1)
			MB>Jet ski (4)
			Jet ski = MB (11)
MB vs Airboat	[119]	9	Airboat>MB (9)

Comparisons were excluded when they were not explicitly tested or described, or when it was unclear if single or multiple walkers were used. Stimuli examined in these studies are single walker (SW), multiple walkers (MW), single jogger (Jogger), single leashed or unleashed dog (Dog), car, truck, motorised boat (MB), non-motorised boat (NMB), jet ski, and airboat.

^1^ Number of comparisons is greater than number of species in cases where different studies have investigated the comparison in the same species.

We conducted 730 approaches to 39 species of waterbird (Table 3). The mean FIDs for each stimulus type, across those 39 species, were: walker, 67.6±37.5 m; three walkers, 92.3±67.7 m; bicycle, 67.7±37.1 m; car, 59.5±37.7 m; and bus, 81.2±96.5 m.

**Table 3:** Flight-Initiation Distances (FID) of 39 species in response to up to five stimuli.

	Single Walker			Multiple Walker			Bicycle			Car			Bus		
Species (*family*)	FID (M±SD)	StartD (M±SD)	n	FID (M±SD)	StartD (M±SD)	n	FID (M±SD)	StartD (M±SD)	n	FID (M±SD)	StartD (M±SD)	n	FID (M±SD)	StartD (M±SD)	n
***Anatidae***															
Musk Duck *Biziura lobata*	55.6±24.0	111.2±40.4	2				70.4	112.6	1	38.7±24.6	132.8±77.9	2			
Cape Barren Goose *Cereopsis novaehollandiae*	82.6±40.3	119.6±58.5	5	105.0	261.0	1	102.0±59.7	187.3±59.3	3						
Black Swan *Cygnus atratus*	47.9±20.0	84.7±60.3	18	97.3±71.2	114.0±81.1	6	59.8±67.2	134.2±77.7	6	24.2±14.9	94.9±35.1	5	57.0±47.3	121.4±65.4	14
Australian Shelduck *Tadorna tadornoides*	111.5±53.6	213.5±111.7	21	138.9±83.1	300.6±171.2	22	106.1±44.8	665.9±436.5	11	93.3±43.3	265.1±155.2	32	219.7±183.8	737.4±515.7	20
Pink-eared Duck *Malacorhynchus membranaceus*	65.0±27.4	93.8±62.7	11	98.0±37.1	156.5±85.9	4	56.3±13.1	89.4±24.2	3	92.1	125.4	1	47.2±5.8	188.5±47.6	5
Grey Teal *Anas gracilis*	82.5±5.0	90.7±9.5	2				74.3±17.0	208.9±107.7	3	68.3	300.0	1	82.0±19.5	130.8±42.3	2
Chestnut Teal *Anas castanea*	76.9±20.5	132.0±65.7	28	85.4±51.5	153.8±92.0	18	69.0±24.6	150.2±73.4	34	53.5±20.3	121.7±53.3	24	63.1±30.2	155.5±63.1	20
Pacific Black Duck *Anas superciliosa*	97.9±5.9	245.7±91.8	6	46.0	50.0	1	40.3±29.2	63.5±38.2	3	52.0±24.3	135.4±74.4	14	50.4±23.8	93.2±55.1	4
Hardhead *Aythya australis*	88.4±47.2	157.0±92.8	13	41.0	115.0	1				60.6±24.1	112.6±50.0	9			
Blue Billed Duck *Oxyura australis*	40.0	44.1	1												
***Podicipedidae***															
Australasian Grebe *Tachybaptus novaehollandiae*	55.3	77.9	1							17.7	30.5	1			
Hoary-headed Grebe *Poliocephalus poliocephalus*							16.6	23.6	1						
Great Crested Grebe *Podiceps cristatus*													70.0	102.5	1
***Anhingidae***															
Australasian Darter *Anhinga novaehollandiae*	77.4±0.6	108.5±19.0	2				47.5	128.2	1						
***Phalacrocoracidae***															
Little Pied Cormorant *Microcarbo melanoleucos*	50.6±31.4	94.3±52.7	33	61.0±47.7	99.5±49.6	17	47.3±12.4	111.1±43.9	14	30.8±13.8	104.2±56.5	14	57.6±32.8	123.2±61.0	10
Great Cormorant *Phalacrocorax carbo*	77.9±25.4	89.5±32.9	4							17.7	101.3	1			
Little Black Cormorant *Phalacrocorax sulcirostris*	57.3±69.5	119.1±106.7	5	76.2±69.7	98.7±76.9	8	48.2±21.9	94.5±54.1	7	36.6±21.2	100.2±61.0	7			
Pied Cormorant *Phalacrocorax varius*	72.7±48.9	140.3±84.0	9				128.8±2.1	228.9±32.6	2	62.4	186.8	1			
***Pelecanidae***															
Australian Pelican *Pelecanus conspicillatus*	82.2±15.4	145.4±15.1	4				56.0	109.7	1	99.6±82.9	116.1±82.2	2	95.4±54.8	207.8±60.9	3
***Ardeidae***															
White-necked Heron *Ardea pacifica*	63.4±7.6	71.2±2.5	2							26.4	116.7	1			
Eastern Great Egret *Ardea modesta*	57.2±30.9	89.3±53.5	14	57.7	61.1	1	58.3±10.2	124.6±36.2	5	36.7±17.2	80.1±19.4	3	70.0±8.8	99.5±21.6	2
Intermediate Egret *Ardea intermedia*										20.0	210.0	1			
Cattle Egret *Ardea ibis*	23.4	26.9	1												
White Faced Heron *Egretta novaehollandiae*	45.9±20.4	75.6±37.3	7							44.7±8.1	76.2±7.8	2	81.4	117.6	1
Little Egret *Egretta garzetta*	35.0	39	1												
***Threskiornithidae***															
Australian White Ibis *Threskiornis molucca*	49.8±26.8	94.9±50.2	8							55.1±22.4	109.6±40.0	8	64.9±43.0	194.1±172.2	6
Straw Necked Ibis *Threskiornis spinicollis*	110.7±5.0	138.0±3.6	3							80.6±33.2	261.7±214.0	8			
Royal Spoonbill *Platalea regia*	50.1±29.5	75.6±45.5	8	32.7	80.8	1				46.0±34.9	56.0±26.4	2			
Yellow-billed Spoonbill *Platalea flavipes*	24.7	38.4	1										89.8	149.4	1
***Rallidae***															
Purple Swamphen *Porphyrio porphyrio*	52.6±18.5	77.7±33.1	13	72.5±62.1	113.3±76.9	6	69.7±71.2	103.6±57.9	4	43.5±34.5	92.9±30.4	13	66.9±75.8	139.6±128.9	19
Buff-banded Rail *Gallirallus philippensis*													14.6	23.3	1
Black-tailed Native-hen *Tribonyx ventralis*	52.7±16.8	85.0±40.2	6				35.0	37.0	1				96.0	117.0	1
Dusky Moorhen *Gallinula tenebrosa*										12.0	14.0	1			
Eurasian Coot *Fulica atra*	74.7±34.9	96.2±31.9	4				97.2	112.2	1	74.3±47.6	142.0±74.8	14	62.3±19.2	123.4±41.3	7
***Recurvirostridae***															
Black-winged Stilt *Himantopus himantopus*													19.7	74.4	1
Red-necked Avocet *Recurvirostra novaehollandiae*													56.9±5.7	156.7±42.5	2
***Charadriidae***															
Masked Lapwing *Vanellus miles*													49.9±46.8	149.8±77.0	9
***Scolopacidae***															
Sharp-tailed Sandpiper *Calidris acuminata*													35.4±32.2	89.9±45.4	11
***Laridae***															
Whiskered Tern *Chlidonias hybrida*													46.5±48.2	141.2±135.9	3

The mean ± SD FID (m) and Starting Distance (StartD; m) of 39 species of waterbirds found at WTP in response to approaches by five stimuli: single walker, multiple walker, bicycle, car, and bus. Blanks indicate an absence of data. Species are ordered by family following the taxonomy of Christidis and Boles [122].

**Figure 1:**
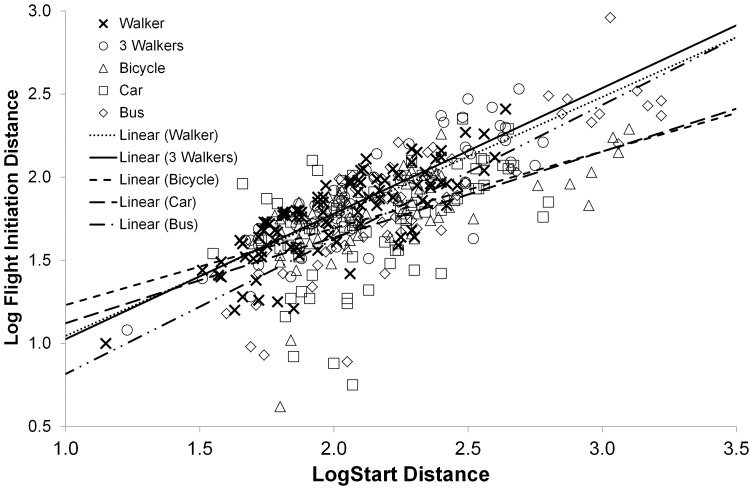
The relationship between Flight-Initiation and Starting Distance for each stimulus type. Data are from four species that had at least five FIDs for each stimulus type (black swan, Australian shelduck, chestnut teal and little pied cormorant). Symbols: single walker (X), three walkers (○), bicycle (▵), car (□) and bus (◊).

**Figure 2:**
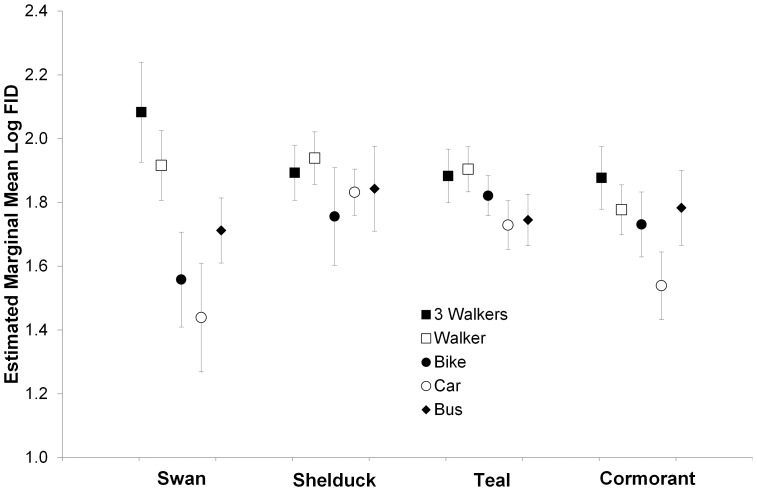
Estimated marginal means for the Flight-Initiation Distance of four species (black swan, Australian shelduck, chestnut teal and little pied cormorant) in response to five stimulus types. Figures are derived from a General Linear Model which revealed a significant interaction between species and stimulus type. Values are estimated marginal means ± 95% C.I.

Within the four species where we had at least five estimates of FID for each stimuli, Starting Distance was positively correlated with FID (*F*_1,339_ = 233.10; *P*<0.001). However, Starting Distance differed between species (*F*_3,363_ = 61.81; *P*<0.001) and stimulus type (*F*_4,367_ = 6.99; *P*<0.001) and the relationship between Starting Distance and FID varied between stimulus types (*F*_4,339_ = 2.60; *P* = 0.036; Figure 1) and between species (*F*_7,339_ = 5.11; *P* = 0.002). There was also a significant interaction between stimulus type and species (*F*_12,339_ = 3.17, *P*<0.001; Figure 2). These results suggested that comparisons between stimuli would be best made on a species by species basis.

To explore the species-specific patterns, we conducted ANCOVA for each species where five or more estimates of FID for at least two stimuli were available (Table 4). Of the 60 pairwise comparisons, 43% [26] revealed significant differences. While most analyses had observed power greater than 0.500, power was quite low in some species (Table 4) and results in these species must be treated with caution. Seven of the twelve species discriminated between stimuli (i.e. had at least one significant pairwise difference; 1–10 comparisons across taxa), but often the effect size was modest (see, for example, Figure 2). FIDs differed between all possible comparisons between stimuli in at least one species (Table 4). Single and multiple walkers evoked longer FIDs than cars (10 of 15 pairwise comparisons; 5 comparisons reported no difference) and buses (4 of 6 significant comparisons; 6 comparisons reported no difference; Table 4). Thus, of the 16 significant comparisons between humans on foot and motor vehicles, humans evoked longer FIDs in 14 comparisons (88%). Pedestrians, singly or in groups, also evoked longer FIDs than bicycle riders in most cases (4 of 5 significant comparisons; 6 comparisons reported no difference). The number of comparisons between cars, buses and bicycles were too few to permit any generalisations, although two (of 2) comparisons involved shorter FIDs to cars compared with bicycles.

**Table 4:** Summary of pairwise comparisons of FID (logged) for analyses of each species across stimulus types (i.e. where ≥5 replicates were obtained for any stimulus type).

Species	Stimulus	3 Walkers	Bicycle	Bus	Car
Australian shelduck (4/10) [0.816]	Bicycle	CYC<MW *			
	Bus	NS	NS		
	Car	CAR<MW**	NS	NS	
	Walker	NS	SW>CYC *	NS	CAR<SW *
Black swan (6/10) [0.996]	Bicycle	CYC<MW ***			
	Bus	BUS<MW ***	NS		
	Car	CAR<MW ***	NS	NS	
	Walker	NS	SW<CYC ***	SW<BUS *	SW>CAR **
Chestnut teal (7/10) [0.992]	Bicycle	NS			
	Bus	BUS<MW **	BUS>CYC *		
	Car	CAR<MW **	CAR<CYC *	NS	
	Walker	NS	SW>CYC *	SW>BUS ***	SW>CAR ***
Little pied cormorant (4/10) [0.980]	Bicycle	NS			
	Bus	NS	NS		
	Car	CAR<MW ***	CAR<CYC **	CAR<BUS **	
	Walker	NS	NS	NS	SW>CAR ***
Australian white ibis (0/3) [0.148]	Bicycle				
	Bus				
	Car			NS	
	Walker			NS	NS
Pink-eared duck (1/1) [0.767]	Bicycle				
	Bus				
	Car				
	Walker			SW>BUS *	
Eastern great egret (0/1) [0.076]	Bicycle				
	Bus				
	Car				
	Walker		NS		
Purple swamphen (2/6) [0.618]	Bicycle				
	Bus	NS			
	Car	NS		NS	
	Walker	NS		SW<BUS *	SW>CAR *
Pacific black duck (0/1) [0.511]	Bicycle				
	Bus				
	Car				
	Walker				NS
Eurasian coot (0/1) [0.050]	Bicycle				
	Bus				
	Car			NS	
	Walker				
Little black cormorant (2/6) [0.806]	Bicycle	NS			
	Bus				
	Car	CAR<MW **	NS		
	Walker	SW<MW *	NS		NS
Hardhead (0/1) [0.258]	Bicycle				
	Bus				
	Car				
	Walker				NS
Overall (26/60)	Bicycle	2/5			
	Bus	2/5	1/4		
	Car	5/6	2/5	1/7	
	Walker	1/6	3/6	4/7	5/9

Brackets after the species name refer to the number of significant comparisons (out of the comparisons conducted). Square brackets refer to the observed power of the analysis. Single walker (SW), multiple walkers (MW), bicycle (CYC), car (CAR) and bus (BUS).

Blanks indicate no comparison was possible, ‘NS’ is not significant, ‘*’ means *P*<0.05, ‘**’ means *P*<0.01 and ‘***’ means *P*<0.001.

## Discussion

The majority of FID studies focus on a single stimulus, usually a single walker. The few studies which have compared species response across more than one stimuli have found that while some species discriminate between stimuli, many do not. Where we report no difference between stimuli with regard to FID we acknowledge that low power sometimes existed, thus the cases where we report a lack of difference between stimuli should be treated with caution. The available dataset for determining meaningful buffers for non-walker stimuli around sensitive sites relies on data from single walkers. This study suggests that such buffers will often also effectively protect against most disturbance by the other stimuli we tested, at least at the study site and for the species studied. However, we report at least one case where buses, bicycles and multiple walkers evoked longer FIDs than single walkers, and we caution against the use of “walker-only” FIDs in all cases. Any elucidation of general principles regarding the influence of stimulus on bird response is clearly to be encouraged.

This study suggests that some but not all species discriminate among the stimuli we tested. Some birds have the capacity to discriminate between stimuli in terms of their responses [4], [9], and are even capable of discriminating between behaviour of the same stimulus [23], [24]. Many studies of discrimination between stimuli focus on a single species [10], [25], [26], [27] but multi-species studies ([9]; this study) report species differences in the capacity to discriminate between stimuli, with some species not adjusting responses between different stimuli. While this may result from low statistical power, or because the stimuli presented are similarly threatening and so responses are equivalent, it may also mean some species do not discriminate between stimuli and instead generalise their response to a variety of perceived anthropogenic threats. Discrimination between stimuli is expected to evolve where a fitness advantage is derived from such discrimination, or where species have the capacity to learn to adjust their responses [28].

Although species varied in their response to different stimuli, this study confirms that, where discrimination between stimuli occurs, vehicles tend to evoke shorter FIDs than humans on foot. This has previously been observed in some [29] but not all species examined [30]. We are unaware of any previous studies using buses as stimuli, and while for six species cars and buses evoked the same FIDs, for one species buses evoked longer FIDs than cars. Single and multiple walkers evoked the same FIDs in five of six species; human group size is rarely studied though has been proposed as a factor which might mediate FID [4]. We are aware of only three studies that have examined the influence of human group size on FID [8], [31], [32]. Lee *et al.* [31] and Kerbitou *et al.* [32] found no effect of human group size on FID, while Geist *et al.* [8] found one of two species distinguished between human group size. As for all studies of this type, the generalizability of the specific stimuli we used is unknown. For example, larger buses, noisier, speedier or different coloured cars, may influence responses. The fundamental attributes of stimuli which are used by birds to adjust responses remain unknown and represent a tantalising prospect for an experimental study [4].

A major aim of this study was to examine whether management of stimuli could reduce disturbance to waterbirds. While vehicles sometimes but not always reduce FIDs, they can carry a number of humans (5–7 for cars; the bus we used could carry 25 passengers). Thus, on a per human basis, vehicles dramatically reduced the response of waterbirds to humans compared with the situation where humans walked singly through the site. However, vehicles can travel greater distances than walkers over the same time frame, potentially exposing more birds to vehicles. Indeed, vehicles may reach areas effectively unreachable by walkers (and vice versa). Ultimately, in large wetlands such as the one we studied, the frequency with which birds are affected by disturbance will be influenced more by the capacity to carry numbers of people and the distance covered by the different modes of transport, than by the FIDs each transportation mode evokes.

Overall, our results demonstrate that at least some species can differentiate between stimuli, with motor vehicles apparently being less disturbing than pedestrians. However, when managing disturbance, it is very important to establish the extent of access and likely occurrence of humans on foot versus vehicles, the frequency of occurrence of each stimulus type, and how the distribution of each stimulus overlaps with important habitat used by birds.

## Supporting Information

Figure S1 **PRISMA flow diagram describing the literature search and selection of articles for analysis.**
(DOC)
