# Supplementary material for: Buses, Cars, Bicycles and Walkers: The Influence of the Type of Human Transport on the Flight Responses of Waterbirds
Source: PLoS One. 2013 Dec 18;8(12):e82008. doi: 10.1371/journal.pone.0082008 (PMC3867343; doi:10.1371/journal.pone.0082008)
Supplement: Figure S1 — PRISMA flow diagram describing the literature search and selection of articles for analysis. (DOC) [file pone.0082008.s001.doc]

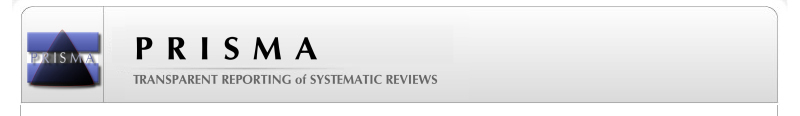
**PRISMA 2009 Flow Diagram**

**Screening**

**Included**

**Eligibility**

**Identification**

Records identified through database searching
(n =695 )

Additional records identified through other sources
(n =0 )

Records after duplicates removed
(n =695 )

Records screened
(n =695 )

Records excluded
(n =595 )

Full-text articles assessed for eligibility
(n =100 )

Full-text articles excluded, with reasons
(n = 0 )

Studies included in qualitative synthesis
(n = 0 )

Studies included in quantitative synthesis (meta-analysis)
(n =100 )
